# Diversity, equity and inclusion considerations in the design of digital mental health interventions for psychosis: A scoping review^☆^

**DOI:** 10.1016/j.invent.2026.101013

**Published:** 2026-10-28

**Authors:** Thomas Gant, Kathryn M. Taylor, Thomas Ward, Philippa Garety, Amy Hardy

**Affiliations:** a Institute of Psychiatry, Psychology & Neuroscience, King's College London, London, United Kingdom; b South London & Maudsley NHS Foundation Trust, London, United Kingdom

**Keywords:** mHealth, Digital therapeutics, Psychotic, Equality, Accessibility, Co-design, Inclusive design

## Abstract

Digital mental health interventions (DMHIs) are increasingly used to support people with psychosis. However, little is known about how diversity, equity, and inclusion (DEI) are considered in their design. In this review, DEI was operationalised as design considerations relating to access, content, appearance, and stakeholder involvement, employed to support development of equitable and inclusive DMHI, and evaluated using an adapted Culturally Informed Design Framework. While stakeholder engagement is frequently reported, the extent to which design processes systematically address DEI domains remains unclear. This scoping review examines what design approaches have been used, the nature of stakeholder involvement, and the DEI domains considered in DMHI development for psychosis. A systematic search across six databases identified 167 eligible studies, describing 55 DMHIs. Data were extracted and synthesised. Thirteen interventions (23.6%) explicitly named a design approach, with user-, person-, or human-centred design most frequently reported. Thirty-one interventions (56.4%) reported some level of stakeholder engagement in design, most commonly involving people with lived experience of psychosis and clinicians. Forty-three interventions (78.2%) were reported as addressing at least one DEI-related feature. At the subdomain level, literacy (n = 15), system requirements (n = 16), psychosis-specific adaptations (n = 14), and diverse visual representation (n = 13) were among the most frequently reported considerations, whereas cultural adaptation (n = 3), availability in languages beyond English (n = 3), and environmental tailoring (n = 5) were rarely reported. Interventions characterised by sustained stakeholder engagement across multiple stages of development tended to demonstrate broader DEI coverage, although inclusion was rarely articulated as an explicit design goal. These findings highlight that while participatory design approaches are commonly employed, published reports indicated limited and uneven integration of DEI considerations in DMHI design. To support equity and inclusion, DMHI development should adopt a theoretically-informed design approach with stakeholder involvement throughout and comprehensive targeting of DEI domains.

## Highlights

• Little is known about how equity is embedded in psychosis intervention design.
• Scoping review synthesised 167 studies describing 55 interventions.
• Most addressed one equity domain; multi-domain integration was rare.
• Sustained stakeholder involvement broadened equity coverage.
• Structured equity frameworks should guide intervention design and development.

## 1 Introduction

Digital mental health interventions (DMHIs) can support people in managing symptoms of psychosis by delivering information, support, or therapy through technology-based platforms (Hollis et al., 2017; Torous et al., 2021; Chivilgina et al., 2021; Sawyer et al., 2023; Jameel et al., 2022). With increasing device ownership among people with psychosis and growing service user interest, investment in DMHIs is expanding (Xia et al., 2025). Evidence supports their feasibility, acceptability, and preliminary efficacy (Morales-Pillado et al., 2023; Camacho et al., 2019; Clarke et al., 2019). DMHIs therefore hold promise for providing more accessible, scalable, and effective care for people with psychosis, particularly for underserved communities who face barriers to traditional care (Lattie et al., 2022; Haddock et al., 2014; Morris et al., 2020).

Despite their potential, engagement with DMHIs is often poor, and implementation success remains limited (Bennion et al., 2017; Borghouts et al., 2021). One explanation is that many DMHIs are designed using top-down approaches, where experts draw primarily on evidence-based strategies and neglect the lived experience of intended users (Mohr et al., 2017). As a result, interventions may fail to reflect the contexts and needs of individuals (Naslund et al., 2017). This problem is compounded by the systematic underrepresentation of marginalised groups in digital health research, including people from racialised communities, those with lower education and socioeconomic status, older adults, disabled people, and those living in rural areas (World Health Organization, 2019; WHO, 2022).

These inequities are particularly concerning given that psychosis disproportionately affects individuals from minoritised backgrounds. Incidence rates are two to five times higher among people from Bangladeshi, Pakistani, and Black ethnic groups compared with White British populations (Kirkbride et al., 2017), and psychosis is strongly associated with social deprivation (Jongsma et al., 2021). People with psychosis may face distinct challenges in using technology, including cognitive difficulties and elevated concerns about privacy (Palmier-Claus et al., 2013; Rajapakse et al., 2011). These challenges intersect with broader inequities in digital access and literacy (termed the “digital divide”) which disproportionately affect underserved groups (Robotham et al., 2016; Spanakis et al., 2021; Watson et al., 2022). DMHIs for psychosis risk perpetuating or exacerbating existing health disparities if not designed to promote inclusion.

Diversity, equity, and inclusion (DEI) encompass related but distinct considerations (Mishra et al., 2025). Diversity refers to variation across individual characteristics such as age, gender, sexuality, race, ethnicity, nationality, religion, education, livelihood, and marital status (American Psychological Association, 2020). Equity concerns the absence of unfair, avoidable, or remediable differences between groups (World Health Organization, 2025). In digital health, inclusion concerns whether individuals and communities, including those who may be disadvantaged, can access and effectively use digital technologies, including through efforts to address historical, institutional, and structural barriers to their use (National Digital Inclusion Alliance, 2021). Embedding DEI considerations within DMHI design may help to reduce healthcare inequities and aligns with global public health priorities (Robinson et al., 2024; WHO, 2019). Within digital mental health, cultural adaptation has received increasing attention, with adaptations spanning intervention content, methodology, and development processes (Spanhel et al., 2021). Evidence suggests that culturally relatable DMHIs can enhance engagement and may improve effectiveness (Bakhti et al., 2025). Within psychosis, emerging research has similarly highlighted the importance of inclusive design. Design features such as clear and simple language, support for low literacy levels, and diverse and representative visual assets have been identified as important facilitators of engagement (Eisner et al., 2025). Given that engagement is a critical mechanism influencing the effectiveness of digital interventions, incorporating DEI in design may be a driver for optimising impact (Graham et al., 2021).

Design approaches provide principles and methods for engaging underserved populations and addressing diverse needs within DMHI development (Lwembe et al., 2017). A recent systematic review of co-production in DMHI development found that end users were rarely involved across all stages of development, while reported benefits of co-production included greater intervention acceptability and cultural sensitivity (Brotherdale et al., 2024). Within psychosis, meaningful involvement of people with lived experience and co-production have been identified as priorities for digital intervention development (Smith et al., 2024). This underscores the importance of examining both the design approaches employed and the nature of stakeholder involvement in DMHI development. However, terminology in this area (e.g., patient and public involvement (PPI), co-production, co-design, and user- or human-centred design) is often used interchangeably and without clear definition (Baines and Regan de Bere, 2018; Vial et al., 2022). Distinctions between these approaches are important. For example, co-production typically involves stakeholders in implementing a previously defined problem and solution to improve outcomes, whereas in co-design, stakeholder engagement shapes the identification of the problem itself and the development of corresponding solutions (Vargas et al., 2022).

Several frameworks have been developed to support the evaluation of digital health interventions (e.g., National Institute for Health and Care Excellence, 2022a; Stoyanov et al., 2015). However, while these frameworks capture aspects relevant to DEI, such as accessibility, usability, and cost, DEI is not typically their primary focus, resulting in limited coverage (Ramos et al., 2021). This variation presents a challenge for systematically addressing and evaluating DEI in DMHI design, highlighting the need for an explicit framework through which DEI considerations can be operationalised. The Culturally Informed Design Framework (CIDF; Valdez et al., 2012; Ramos et al., 2021; Figueroa et al., 2024) provides one such approach, considering DEI domains across multiple aspects of intervention design, including access, content, appearance, and stakeholder involvement. Rather than treating DEI as a single characteristic of an intervention, the framework enables consideration of how different needs, characteristics, and barriers may be addressed across the design process.

Despite increasing attention to DEI in digital health, it remains unclear how these considerations have been incorporated into the design of DMHIs for psychosis. This review will therefore employ the CIDF as a structured means of operationalising DEI, to evaluate how it is addressed in the design of DMHI interventions for psychosis. Given the review focus, the CIDF will be adapted to capture inclusivity considerations specific to the needs and experiences of people with psychosis. In this review, design is conceptualised as an iterative process of intervention development, including formative research, prototyping, refinement, and post-pilot modification. This scoping review addresses this gap by systematically mapping existing evidence and identifying opportunities to support the development of inclusive DMHIs for psychosis that deliver equitable outcomes.

The research questions guiding this review are:

1. What design approaches have been used in the development of DMHIs for psychosis?
2. What stakeholders have been involved, and how have they been involved?
3. Which DEI-related domains are reported as being addressed in the design of DMHIs for psychosis?

## 2 Methods

### 2.1 Study design

This scoping review was guided by the Joanna Briggs Institute Scoping Review Methodology (Peters et al., 2020) and conducted and reported following the Preferred Reporting Items for Systematic Reviews and Meta-Analyses extension for Scoping Reviews (PRISMA-ScR; Tricco et al., 2018). The protocol for the scoping review was prospectively registered at the Open Science Framework (doi:10.17605/OSF.IO/NRH6B).

### 2.2 Eligibility criteria

Eligibility criteria were developed using the population, concept, and context (PCC) framework. Studies were included if they:

(a) were aimed at individuals aged 16 years or older with a primary diagnosis of schizophrenia-spectrum disorder (SSD) (ICD-10; WHO, 2019: F20–F29). In instances of diagnostic heterogeneity, more than 50% of the total sample was required to have a primary diagnosis of SSD;
(b) examined a digital mental health intervention (DMHI; defined according to Hollis et al., 2017) with a primary focus on the psychological symptoms, mechanisms, or sequelae of psychosis. This included interventions targeting associated difficulties, such as anxiety or agoraphobia, where these were specifically addressed in the context of psychosis; and
(c) were peer-reviewed, available in English, and published within the last ten years (on or after 1 February 2015), reflecting the rapid evolution of digital technologies.

Articles were excluded if the DMHI was aimed solely at carers or targeted neurocognitive symptoms associated with psychosis (e.g., social cognition deficits, memory impairment, attentional difficulties). Evidence syntheses (e.g., systematic reviews, meta-analyses) and conference abstracts were excluded.

### 2.3 Information sources & search strategy

Six electronic databases were searched in February 2025: EMBASE, Medline, PsycINFO, ProQuest, SCOPUS, and Web of Science. Search terms were developed using the PCC framework and focused on SSD, digital mental health interventions (e.g., virtual reality, web-based platforms, mobile applications), and intervention type (e.g., self-help, CBT). Full search strings for each database are presented in Supplementary File 1.

Consistent with the aims of a scoping review, the search strategy was intentionally broad and inclusive of all study designs. This approach was adopted because information on intervention design, stakeholder involvement, and DEI-related features is rarely confined to standalone design papers and is often distributed across protocols, feasibility studies, qualitative evaluations, and implementation reports. Including all peer-reviewed publications linked to each intervention enabled a more comprehensive mapping of design practices.

To support comprehensive collection of design-related information, earlier (pre-2015) papers reporting on intervention design processes were identified through hand searching the reference lists of included full-text articles, as these publications would not have been captured by the database searches. Data from these earlier publications were extracted where they related to interventions that otherwise met the inclusion criteria.

Given the challenges of systematically identifying and reproducing grey literature searches, the review was limited to peer-reviewed sources to maintain feasibility, consistency, and transparency.

### 2.4 Selection of sources

Rayyan software (version 1.5.0) was used for title and abstract screening and full text screening. All titles and abstracts were screened by the first author and marked as “include”, “exclude”, or “maybe”. A random 15% of titles and abstracts were independently screened by co-author KT, and Cohen's Kappa for inter-rater agreement was near perfect (0.90). All full text articles were screened by first author and marked as “include” or “exclude”. Reasons for exclusion were noted based on the eligibility criteria. A random selection of 40% of full text articles were also screened by co-author KT. Cohen's Kappa was again near perfect (0.90). Disagreements were resolved through consensus between the two reviewers. Consistent with JBI guidance for scoping reviews (Peters et al., 2020), formal critical appraisals of study quality were not undertaken, as the aim was to map the extent and nature of available evidence.

### 2.5 Data extraction and synthesis

An extraction table was developed by the lead author using Microsoft Excel and iteratively refined in consultation with co-authors. Data were extracted into three datasets.

The first dataset captured key characteristics of the included interventions, including: (a) intervention name; (b) digital modality; (c) therapeutic target (e.g., hallucinations, delusions); (d) delivery setting(s); (e) country or countries of development and evaluation; (f) study design(s); and (g) citation(s).

The second dataset examined the design approaches reported in the development of DMHIs, the stakeholder groups involved, and design methods. Design approaches were recorded only where explicitly named by study authors and were not inferred from the methods described. Where no formal approach was named, reported formative or developmental activities were recorded where they directly informed intervention development or refinement, such as stakeholder consultation, needs assessment, prototyping, usability testing, or iterative modification.

The third dataset mapped DEI-related domains reported as being addressed in DMHI design. DEI considerations were operationalised using the adapted Culturally Informed Design Framework (Valdez et al., 2012; Ramos et al., 2021; Figueroa et al., 2024). The adapted framework comprised four domains: (a) access requirements; (b) content; (c) appearance; and (d) stakeholder involvement, with predefined subdomains within each (Supplementary File 2).

To capture inclusivity in relation to the needs and experiences of people with psychosis, content-related considerations of disability and specific needs within the framework were extended to include psychosis-specific tailoring. This captured design adaptations to address symptoms or difficulties associated with psychosis that may affect engagement with a digital intervention. Examples included visual cues to support cognitive difficulties and providing users with control over whether technologies were linked where data sharing or connectivity could exacerbate paranoia-related concerns. Psychosis-specific tailoring was coded only where a specific design feature or adaptation addressing a psychosis-related need was reported; development of an intervention for people with psychosis was not itself sufficient for this subdomain to be coded.

A DEI-related subdomain was coded as addressed where the published literature explicitly reported a corresponding design feature, adaptation, or design activity. Discussion of a DEI consideration without evidence that it informed intervention design was not sufficient for the subdomain to be coded. The framework was applied as an analytic lens to categorise reported features of an intervention's design, rather than to infer author intent or retrospectively evaluate the quality or adequacy of original design decisions. Where DEI-related features were not reported, this was treated as absence of evidence rather than evidence of absence.

## 3 Results

### 3.1 Selection of sources

A total of 51,708 records were identified through database searches. After removal of duplicates, 36,533 records were screened based on titles and abstracts. Of these, 215 full-text articles were assessed for eligibility, resulting in 150 studies included in the final synthesis. An additional 17 studies were identified through hand searching of the reference lists of included studies, yielding a total of 167 studies for final inclusion. The study selection process, including reasons for exclusion at the full-text stage, is presented in the PRISMA flow diagram (Fig. 1).

**Fig 1:**
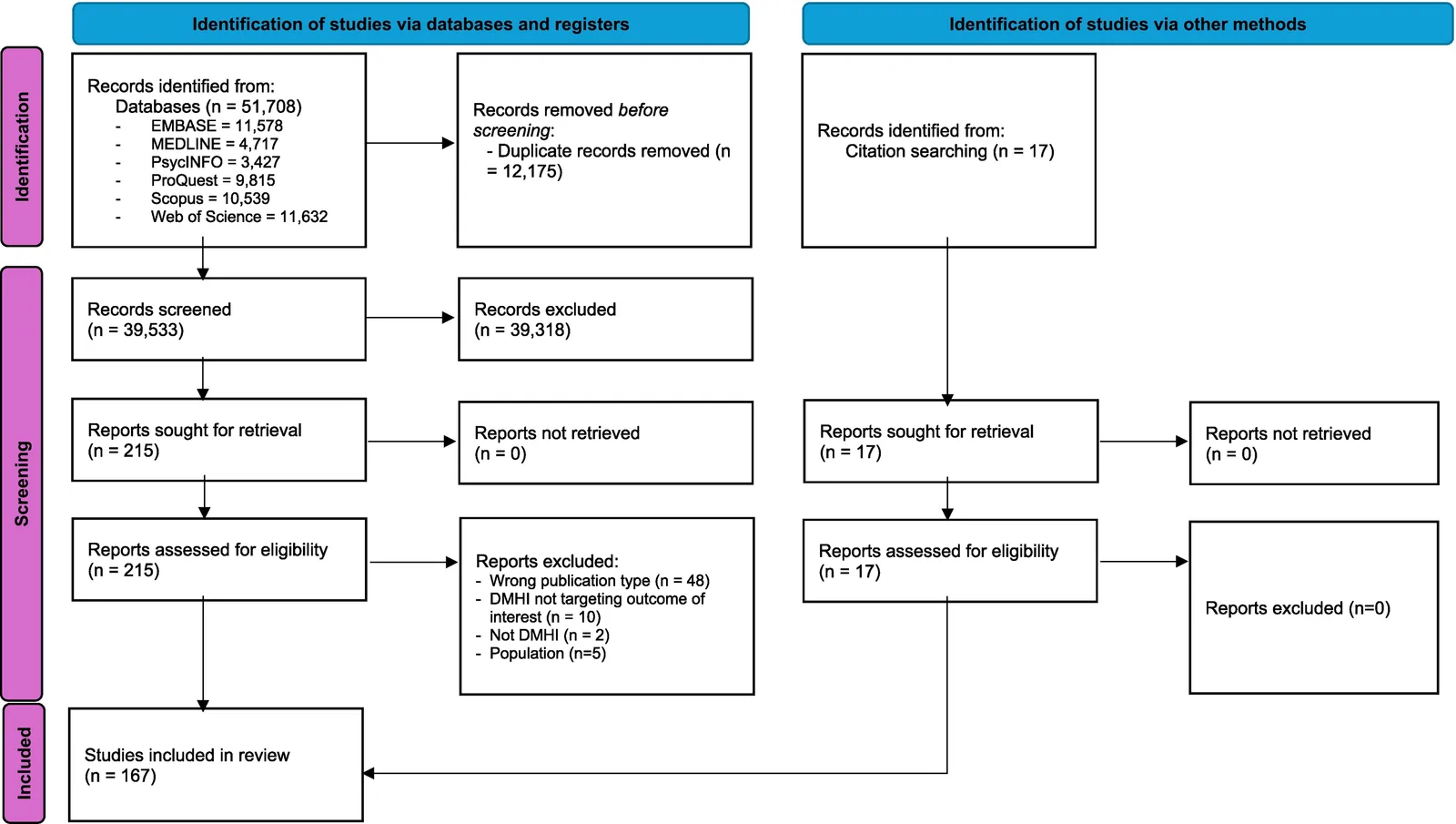
PRISMA flow diagram of search and study selection process.

### 3.2 Characteristics of DMHIs for psychosis

A summary of the included digital mental health interventions (DMHIs), including their digital modality, therapeutic target, setting, country, design and citations are presented in Table 1. Detailed descriptions of individual interventions are provided in Supplementary File 3. Across the 167 included studies, 55 distinct DMHIs for psychosis were identified. Interventions were developed and evaluated across a range of geographical contexts, with the majority originating from Europe (n = 33), followed by North America (n = 11). Fewer interventions were developed in Asia (n = 4) and Australia (n = 3). Four interventions were developed across multiple continents.

**Table 1:** Key characteristics of digital mental health intervention, designs, and settings.

Intervention	Modality	Therapeutic target	Setting(s)	Country(s)	Study design(s)	Citation(s)
A4i	Mobile app	Self-management	EIP service	Canada	Pre-post feasibility study; feasibility RCT protocol; feasibility RCT, secondary analysis; brief report	Kidd et al., 2019, Kidd et al., 2021, Kidd et al., 2025; Price et al. (2022); Sequeira et al. (2023)
Actissist	Mobile app	Self-management	EIP services	United Kingdom	RCT; proof of concept; design paper; RCT; secondary analyses	Bucci et al., 2015, Bucci et al., 2018, Bucci et al., 2024; Berry et al. (2020); Eisner et al. (2023); Hassan et al. (2023)
AVATAR therapy	VR-related therapy	Auditory hallucinations	NHS mental health services	United Kingdom	Proof of concept; development paper; protocols; RCTs; qualitative analyses; cross sectional survey; secondary analyses	Leff et al. (2013)⁎; Huckvale et al. (2013); Craig et al., 2015, Craig et al., 2018; Hall et al. (2018); Rus-Calafell et al., 2020, Rus-Calafell et al., 2022; Ward et al., 2020, Ward et al., 2021; Garety et al., 2021a, Garety et al., 2021b, Garety et al., 2021c, Garety et al., 2024; O'Brien et al. (2021)
Avatar-assisted therapy	VR-related therapy	Auditory hallucinations	Recruitment centres	Spain	Co-design study	García et al. (2021)
CAM+VR	VR	Social anxiety	EIP service	Canada	Case study	Hache-Labelle et al. (2023)
CBT + AT	VR	Auditory hallucinations	Outpatients	Poland	Case study; pilot study	Stefaniak et al., 2017, Stefaniak et al., 2019
CBT2go	Blended	Self-management	Outpatients	USA	RCT	Depp et al. (2019)
Challenge-VRT	VR	Auditory hallucinations	Outpatients	Denmark	Protocol	Smith et al. (2022)
ChillTime	Mobile app	Emotion regulation in dual disorders	Outpatient and inpatients	Canada	Pre-post pilot study	Pennou et al. (2023)
CWV	Web-based	Auditory hallucinations	Outpatients	USA	RCT	Gottlieb et al. (2017)
DICE	Blended	Delusions	Outpatients	Germany	Feasibility trial	Böge et al. (2025)
Digital NAVIGATE	Blended	Psychosis symptoms	NAVIGATE clinic	Israel	Development and single-arm pilot	Cohen et al. (2024)
EMBRACE	Web-based	Social anxiety	PPI; EIP clinic	Australia	Design paper; single-group pilot	McEnery et al., 2019, McEnery et al., 2021
EMPOWER	Blended	Self-management & relapse prevention	Community mental health services	Australia & United Kingdom	Protocols; qualitative studies; feasibility cluster RCT	Allan et al., 2019a, Allan et al., 2019b, Allan et al., 2021, Allan et al., 2023; Gumley et al., 2020, Gumley et al., 2022a, Gumley et al., 2022b
EviBaS	Mobile app	Psychosis symptoms	Former patients diagnosed with SSD, psychiatric institutions, online recruitment	Germany & Switzerland	Protocol; RCT; secondary analysis	Rüegg et al. (2018); Lüdtke et al., 2020, [Bibr bb0760][Bibr bb1140]
FOCUS	Blended	Self-management	Outpatients (recently discharged); VA hospital	USA	Development and usability testing; qualitative studies; feasibility, acceptability and usability studies; secondary analyses; longitudinal study	Ben-Zeev et al., 2013, ⁎, Ben-Zeev et al., 2014, Ben-Zeev et al., 2016; Achtyes et al. (2019); Jonathan et al. (2019); Carpenter-Song et al. (2020); Buck et al. (2022)
gameChange	VR	Agoraphobia	NHS mental health services	United Kingdom	Protocols; therapy development; qualitative studies; cross-sectional survey; RCT; secondary analyses; health economic evaluation	Lambe et al. (2020); Freeman et al., 2019a, Freeman et al., 2022a, Freeman et al., 2022b, Freeman et al., 2023a; Knight et al. (2021); Brown et al. (2020); Bond et al., 2021, Bond et al., 2023; Altunkaya et al. (2022)
HEINS	SMS, mobile app or internet based	Self-management	Outpatients (recently discharged from inpatient)	Germany	Pilot RCT	Gallinat et al. (2021)
HelpID	Web-based	Depression	Former medical centre patients	Germany	Survey & RCT	Moritz et al. (2016)
Horyzons	Blended	Social functioning; vocational recovery; relapse prevention	EIP services	Australia, Canada & USA	Design papers; protocols; qualitative studies; pilot studies; RCTs; secondary analyses; focus groups	Álvarez-Jiménez et al., 2012, Alvarez-Jimenez et al., 2013, Alvarez-Jimenez et al., 2020, Alvarez‐Jimenez et al., 2021; Lederman et al., 2014, ⁎; Lal et al., 2018, Lal et al., 2020, Lal et al., 2021, Lal et al., 2023a, Lal et al., 2023b, Lal et al., 2024; Ludwig et al. (2021); Valentine et al. (2020); O'Sullivan et al. (2022); Engel et al. (2023); Pokowitz et al. (2023)
HYM	Blended	Self-management	EIP service	Korea	Survey study	Kim et al. (2018)
IMPACHS	Mobile & web-based app	Self-management	EIP service	Denmark	Usability study	Austin et al. (2021)
m-RESIST	Blended	Self-management & relapse prevention	Adult mental health services	Hungary, Israel & Spain	Qualitative studies; protocol; feasibility, acceptability and usability study	Huerta-Ramos et al., 2016, Huerta-Ramos et al., 2017; Alonso-Solís et al. (2018); Grasa et al. (2023)
MACS	Mobile app	Self-management	Inpatients (current or recently discharged)	USA	Development and feasibility study; pilot RCT; qualitative study	Moitra et al., 2021, Moitra et al., 2024; Gaudiano et al. (2025)
MCI-S	Mobile app	Psychosis symptoms	Community mental health services	Korea	Quasi-experimental study	Han et al. (2023)
Mindfulness-based training in VR	VR	Psychosis symptoms, mood & cognitive functioning	Day ward & community home for mutual aid	Poland	Pilot study	Plencler et al. (2024)
mindLAMP	Mobile app	Self-management	Focus groups with individuals with lived experience of SMI	India & USA	Qualitative study	Rodriguez-Villa et al. (2021)
Modified AVATAR therapy	VR-related therapy	Auditory hallucinations	Outpatients	Hungary	Case series	Vass et al. (2024)
MUSE-FEP	Blended	Auditory hallucinations	EIP services	United Kingdom	Single-arm feasibility study; protocol; feasibility RCT	Dodgson et al. (2021); Dudley et al., 2022, Dudley et al., 2024
My Journey 3	Mobile app	Self-management	EIP services	United Kingdom	Protocol for feasibility RCT; feasibility study; qualitative study	Steare et al., 2019, Steare et al., 2020, Steare et al., 2021
NEON	Web-based	Recovery	N/A	United Kingdom	Intervention development; study protocol; secondary analysis	Slade et al., 2021, Slade et al., 2024a, Slade et al., 2024b; Rennick-Egglestone et al. (2020); Ali et al. (2024)
PhoenixVR	VR	Psychological wellbeing	NHS mental health services	United Kingdom	Proof of concept; protocol; design paper	Freeman et al., 2023a, Freeman et al., 2024; Rosebrock et al. (2024)
SAVVy	Blended	Auditory hallucinations	Specialist voice clinic + wider advertising to clinical services	Australia	Protocol; development and case illustration; pilot RCT; qualitative analysis	Bell et al., 2018a, Bell et al., 2018b, Bell et al., 2020; Moore et al. (2019)
Sleep Catcher	Blended	Sleep difficulties	N/A	USA	App development and usability testing	Jeon et al. (2024)
Sleepio	Mobile app	Sleep difficulties	EIP services	United Kingdom	Protocol for feasibility study	Beattie et al. (2023)
SloMo	Blended	Paranoia	NHS mental health services	United Kingdom	Experimental investigation; protocol; design papers; qualitative studies; secondary analyses	Garety et al., 2017, Garety et al., 2021a, Garety et al., 2021b; Hardy et al., 2018, Hardy et al., 2022, Hardy et al., 2024; Greenwood et al., 2022a, Greenwood et al., 2022b; Ward et al. (2022); Gant et al. (2025)
SMART	Blended	Self-management	Adult mental health services	Australia	Protocol for RCT; qualitative studies; secondary analyses	Thomas et al. (2016); Williams et al. (2018); Arnold et al., 2019, Arnold et al., 2020
SMARTapp	Mobile app	Self-management	Outpatients and inpatients	Netherlands	Feasibility RCT	Hanssen et al. (2020)
Smartphone app targeting sleep	Mobile app	Sleep difficulties	Community mental health services	United Kingdom	Single-arm feasibility study	Taylor et al. (2022)
START	Blended	Suicidal ideation	Walk-in centre	USA	Pilot trial protocol	Depp et al. (2021)
STOP	Mobile app	Paranoia	People with lived experience of paranoia	United Kingdom	Protocol; RCT; qualitative study; development and usability testing	Yiend et al., 2017, Yiend et al., 2024; Leung et al. (2019); Hsu et al. (2023)
T4Rp	SMS based	Relapse prevention	Focus groups with service users	USA	Therapy development paper	Ybarra et al. (2019)
TechCare	Mobile app	Self-management	EIP services	United Kingdom	Protocol; single arm feasibility study	Husain et al. (2016); Gire et al. (2021)
Temstem	Mobile app	Auditory hallucinations	Specialised mental health organisations	Netherlands	Protocol; RCT	Jongeneel et al., 2018, Jongeneel et al., 2024
THRIVE	VR	Persecutory delusions	NHS mental health services	United Kingdom	Protocol; RCT	Freeman et al., 2019a, Freeman et al., 2019b, Freeman et al., 2022c, Freeman et al., 2023a, Freeman et al., 2023b
V-NeST	VR	Negative symptoms	Community mental health services	United Kingdom	Pilot RCT; EME report	Cella et al., 2022, Cella et al., 2023
VBM	VR	Psychosis symptoms	Outpatient clinic	South Korea	Pilot RCT	Lee et al. (2023)
Virtual reality for persecutory delusions	VR	Persecutory delusions	NHS mental health services	United Kingdom	RCT	Freeman et al. (2016)
VR-CBT	VR	Paranoia	Adult mental health services; EIP & Flexible Assertive Community Treatment facilities	Denmark & Netherlands	Protocol; RCT; secondary analyses	Pot-Kolder et al. (2018); Geraets et al. (2020); Berkhof et al. (2021); Jeppesen et al., 2022, Jeppesen et al., 2025
VR-CBTp	VR	Social difficulties	EIP services	United Kingdom	Case series	Jameel et al. (2025)
VR-SOAP	VR	Social functioning	Adult mental health services	Netherlands	Protocol	Meins et al. (2023)
VRET + CBT	VR	Social phobia	Inpatient service	Poland	Feasibility RCT	Stefaniak and Aleksandrowicz (2024)
VRETp	VR	Social participation	Adult mental health services	Netherlands	Protocol; RCT; secondary analysis; health economic evaluation	Pot-Kolder et al., 2016, Pot-Kolder et al., 2020; Berkhof et al. (2025); van der Stouwe et al. (2024)
VRT	VR-related therapy	Auditory hallucinations	Outpatients	Canada	Pilot trial; case report; RCT; secondary analyses	du Sert et al. (2018); Dellazizzo et al., 2018a, Dellazizzo et al., 2018, Dellazizzo et al., 2020, Dellazizzo et al., 2021; Beaudoin et al. (2021); Hudon et al. (2023)
weCOPE	Mobile app	Self-management	Outpatient community rehabilitation centre	Portugal	Single-arm pilot study	de Almeida et al. (2018)

EIP: early intervention in psychosis; RCT: randomised controlled trial; VR: virtual reality; SMS: short messaging service.

⁎ Studies identified through hand searching that were outside the 10-year publication window but relevant to intervention design.

Digital delivery modalities varied widely. The most common modality was virtual reality (VR) and related technologies (n = 19; e.g., AVATAR, gameChange, THRIVE, Face Your Fears). Followed by mobile applications (n = 15; e.g., Actissist, A4i, TechCare, Temstem), blended interventions combining digital tools with clinician, therapist, or peer support (n = 14; e.g., CBT2go, DICE, Horyzons, HYM, MUSE-FEP), and web-based platforms (n = 4; e.g., CWV, EMBRACE, NEON). One intervention (T4RP) was delivered exclusively via SMS, while two interventions (HEINS and IMPACHS) allowed users to choose between multiple delivery formats (e.g., web-based or mobile application).

Therapeutic targets spanned a broad spectrum, reflecting the heterogeneity of psychosis and its associated psychological sequelae. The most frequently targeted domains were self-management, relapse prevention, and general symptom reduction (n = 24). Several interventions addressed specific psychotic symptoms, including auditory hallucinations (n = 10), paranoia and persecutory delusions (n = 5), and negative symptoms or social functioning difficulties (n = 4). Eleven interventions focused on transdiagnostic or associated difficulties, including sleep disturbance (n = 3), social anxiety (n = 3), emotion regulation (n = 1), suicidal ideation (n = 1), agoraphobia (n = 1), and depression (n = 1).

Of the 167 included studies, 20 focused exclusively on intervention design (i.e. pre-evaluation design and development activities). Most studies involved early-phase evaluations (n = 56), including feasibility, acceptability, and early-phase randomised controlled trials. Thirty-two study protocols were identified. Qualitative or mixed-methods designs (n = 32) and secondary analyses (n = 24) were also common. Only a small number of studies had progressed to phase III RCTs (n = 3). Interventions were most frequently evaluated in community mental health settings (n = 18) and early intervention in psychosis (EIP) services (n = 12), with fewer studies conducted in inpatient or post-discharge settings (n = 4). Eleven interventions were evaluated across multiple services, while ten were evaluated in other settings.

### 3.3 What design approaches have been used in the development of DMHIs for psychosis?

Of the 55 included DMHIs, 31 reported information on design processes and are summarised in Table 2. Thirteen interventions explicitly named at least one formal design approach. Nine reported a single approach, including user-centred design (n = 4), person-centred design (n = 1), iterative design (n = 2), agile methods (n = 1), and participatory design (n = 1). Four interventions reported multiple approaches, including person-centred and participatory design (gameChange), person- and user-centred design (PhoenixVR), user- and human-centred design alongside inclusive design and codesign (SloMo), and user-centred design and codesign (STOP). Eighteen interventions did not explicitly name a design approach; however, most (n = 16) described early-stage or iterative development practices consistent with design activities.

**Table 2:** Stakeholder involvement in the design of digital mental health interventions for psychosis.

Intervention	Stakeholder group(s) involved	Stated design approach	Design methods used
A4i	Case managers; family supports; psychiatrists; service users	Iterative design and development model	• Development: literature, patent & market reviews; focus groups with service users, family supports, psychiatrists, case managers (on paper prototypes). • Iteration & testing: beta testing (individuals with psychosis n = 5), half-day ideation focus group, broader user feedback (n = 38), feasibility RCT (n = 91).
Actissist	EIS clinicians; clinical academics; EIS clinicians; service users; software engineers.	Person-centred design	• Development: service users (n = 21), clinician (n = 48), clinical academics, software engineers focus groups. • Iteration & testing: prototype review & internal beta (engineers n = 5; researchers n = 5), user & clinician beta (service users n = 5; clinicians n = 7), further testing (engineers n = 4; researchers n = 3; clinicians n = 5), post-feasibility interviews (service users n = 14) • Expert reference group (2014–2020): multidisciplinary, met quarterly: lived experience (n = 13), psychiatrists (n = 2), clinical psychologists (n = 2), software engineers (n = 3), service-user researcher (n = 1), academics (n = 2).
AVATAR therapy	Voice hearers; service users	Not stated	• Development & testing: voice hearers (voice/face selection trial, n = 3). • Qualitative evaluation: purposively sampled interviews within RCT (n = 14).
Avatar-assisted therapy	Psychiatrists; engineering team; service users	Not stated	• Service users (n = 29) and psychiatrists (n = 20) collaboratively designed the avatar and assessed utility/usability.
ChillTime	Service users	Not stated	• Development & testing: beta-testing focus groups with service users (n = 4; 1-h app use with quantitative & qualitative feedback), followed by feasibility & acceptability pilot (n = 11).
CWV	Service users	Not stated	• Initial open pilot study (n = 21) measured usage and user satisfaction • Pilot study (n = 19) assessing satisfaction and suggestions for improvement
Digital NAVIGATE	Service users; practitioners	User-centred design	• Development: individual client meetings (unmet needs/challenges), practitioner feedback (group/individual meetings; unmet needs/challenges). • Pilot testing: single group pilot (n = 21) evaluating feasibility, usability, acceptability, and preliminary efficacy.
EMBRACE	Clinicians; young adult fiction writers; cartoonist; young people with lived experience of SAD, FEP and SMI (or any combination)	User-centred design	• Development: focus groups with lived experience (n = 16; 1–2 h) to input on comics; content refined iteratively. • Review & refinement: final comics reviewed by collaborative team (consensus) and presented to young people in two focus groups for further refinement.
EMPOWER	Staff supporting people with psychosis' service users; carers	Not stated	• Development: focus groups with mental health staff (n = 88), service users (n = 21), carers (n = 40; 57 min–2 h 9 min) to explore implementation barriers/facilitators; PPI group guiding message curation & review. • Iterative testing: beta-testing with service users (n = 7; 5 weeks) + qualitative interviews; questionnaire piloting (n = 7; 36.7 days). • Feasibility & evaluation: pilot for feasibility, acceptability, and safety n = 42); 1:1 qualitative interviews within trial (service users n = 16; staff – psychiatric nurses n = 6; carer n = 1).
FOCUS	Service users; practitioners	User-centred intervention development	• Needs assessment: mHealth survey with service users (n = 904) and practitioners (n = 8). • Usability testing: service users (n = 12; 2 hr. lab-based). • Feasibility & evaluation: preliminary efficacy study (n = 33); purposively sampled qualitative interviews (n = 16); feasibility & acceptability study in VA (n = 17).
gameChange	People with lived experience of psychosis; designers; software developers; NHS staff	Person-centred design; Participatory design	• Development: lived experience (LE): focus groups, weekly input, and workshops contributed to VR scenarios, therapeutic tasks, style of VR coach, and software design. • User testing & design contribution: detailed user testing during development; 53 LE participants contributed >500 h (varied recovery stages, tech familiarity, including VR-sceptical participants); 12 design workshops (n = 3–9), 17 individual user testing sessions. • Consultation & iterative refinement: LEAP (n = 10) advised on VR scenarios, characters, scripts, prototyping, usability measures; five workshops with NHS staff across five sites provided therapy feedback.
Horyzons	Computer programmers; health informatics experts; web and graphic designers; professional writers; FEP service users; clinicians	Participatory design	• Development: focus groups with young people with psychosis, service users, clinicians, and usability experts on media, functionality, and support needs; online system developed over 30 months; patient workshops on prototype. • Pilot testing & evaluation: single-arm pilot of initial prototype (n = 20) & • Horyzons USA qualitative study: post-treatment interviews with service users (n = 20) on therapy experience. • HoryzonsCa adaptation study: • Stage 1: sociodemographic & tech-use survey (service users n = 11; clinicians n = 15) and beta web portal feedback. • Stage 2: extended personal exploration of beta site (patients n = 6; clinicians n = 4). • Stage 3: consultation interviews & follow-ups for adaptations (clinicians n = 4; patients n = 2). • HoryzonsCa mixed-methods pilot: pilot study (service users n = 23); focus group evaluation (n = 9).
HYM	Psychiatrists; nurses; social workers; psychologists; software technicians	Not stated	• No details provided
IMPACHS	Researchers/clinicians; technology companies	Not stated	• No details provided
M-RESIST	Service user; informal carers; clinicians	Not stated	• Development: 9 focus groups (3 per pilot country; each group: 1 service user, 1 carer, 1 clinician), 35 individual interviews (service users n = 31; carers n = 4; clinicians n = 4) to assess user needs for mHealth solution. • Feasibility & evaluation: study with service users (n = 39) across 3 national sites assessing feasibility, acceptability, and usability.
MACS	Admin support staff; admin/clinical supervisor; clinicians	Not stated	• Development & evaluation: qualitative interviews on initial software version (N = 18): administrative support staff (n = 2), administrative/clinical supervisors (n = 7), clinicians (n = 9).
mindLAMP	Clinicians; family members; service users	Not stated	• Development & evaluation: focus groups across 3 sites (USA & India) after software demonstration: mental health care providers (n = 52), service users & their family members (n = 75).
MUSE-FEP	Service users	Not stated	• Pilot testing: single-arm study with service users on initial version (N = 24).
My Journey 3	App developers; EIP service users & clinicians; pharmacist; NHS manager; digital health experts	Not stated	• Development: local EIP service users consultation; collaborative group of service users, clinicians, pharmacist, and NHS manager guided app development; adaptation of paper-based self-management intervention with digital health experts. • Usability testing: prototype testing with service users (n = 20); usability tests (n = 6) + individual interviews; 1-month field trial (n = 6) with clinician support + interviews. • Feasibility evaluation: qualitative interviews with clinicians (n = 13) and service users (n = 21) participating in feasibility study.
NEON	LEAP; experts in research and intervention development around recovery narratives, eHealth interventions, peer research and mental health recovery; people with current mental health concerns using MH services	Not stated	• Development: three consultation workshops with LEAP to curate recovery narratives (N = 10); recommendations synthesized by 3 LEAP members; iterative refinement of harm minimisation strategies with LEAP and expert panel. • Evaluation & pilot testing: mixed-methods evaluation of initial recovery narratives (N = 40); feasibility evaluation of NEON prototype and trial procedures (N = 25; content modified post-phase). • Qualitative analysis of trial completers (N = 54) • Evaluation of narrative recommender algorithm (N = 739).
PhoenixVR	YPAG; clinical psychologists; treatment designer; software developers	Person-centred/user-centred design	• Development: meetings with YPAG (N = 14; lived experience of psychosis & low self-confidence), software developers, and research team to develop initial design brief; YPAG feedback on virtual environment, coach, and script. • Usability testing: YPAG (N = 14) and separate PPI group (N = 2). • Pilot evaluation: cohort study with service users (N = 12) across 3 NHS Trusts to test MVP usability.
SAVVy	People with lived experience of hearing voices	Not stated	• Development: LEAP members (N = 4) provided feedback on EMA item wording. • Pilot testing: pilot study of new technology (N = 1).
Sleep Catcher	Engineers; mental health researchers	Agile method	• Development: observations of research visits to service users by engineering team; demonstrations and beta testing within research team.
SloMo	Service users; clinicians, researchers; software developers; innovation design engineers	User-centred/human-centred, inclusive design; co-design	• Development: case series with service users (N = 14; semi-structured interviews), design research (N = 18; observations, interviews with service users N = 6, therapist shadowing), creation of user profiles & mapping of service contexts, workshops to synthesize user needs, prototype demonstrations & low-fidelity user testing. • Pilot & evaluation: RCT (N = 361) informing subsequent development, qualitative analysis of user experience (n = 22), case examples on therapy content (N = 140), observational study on user experience predictors (N = 168; treatment arm). • Iterative co-design & testing: 24 transdisciplinary co-design workshops, qualitative interviews with experts by experience (N = 2), participatory analysis, user testing sessions (N = 32), 3 focus groups with service users (n = 9), think-aloud sessions with therapists (n = 10) and service users (n = 11).
Smartphone app targeting sleep	Service user; non-clinical individuals	Not stated	• Development informed by: Service user with lived experience of psychosis (n = 1) and non-clinical individuals (n = 4) consulted on intervention language, format, content, and functionality.
STOP	LEAP; people with lived experience, clinicians, academics; software designers; members of the public	User-centred development; co-design	• Development & content creation: LEAP group and lived experience experts (n = 18) developed 100 everyday situation examples, provided feedback on readability, suitability, and acceptability, and supported scenario development; members of the public (N = 18) gave feedback on graphics. • Evaluation & usability testing: qualitative study on initial software acceptability (N = 8); 15 raters (8 lived experience experts, 7 clinical psychologists) rated scenarios (paranoia severity, readability, item length); 3 × 2 h LEAP meetings (n = 4–5) reviewed items for final intervention; usability testing and feedback on mobile app by lived experience experts (N = 9).
T4RP	Service users; provider administrators; research team	User-centred approach	• Development: service user focus groups (N = 14) and provider/administrator focus groups (N = 11); content advisory teams (service users n = 11; providers/administrators n = 7) reviewed programme content and text messages. • Testing: alpha testing by research team members.
TechCare	Service users; health professionals	Not stated	• Development: interviews with service users (n = 16) on intervention experience; 2 focus groups with health professionals for feedback and refinement; 2-week test run with service users (N = 4) + 1:1 interviews; iterative refinement with software development team. • Pilot & evaluation: pilot study with service users (N = 12) for further refinement; pre-intervention qualitative interviews (n = 16) and post-intervention interviews (n = 13).
Temstem	Group of voice hearers	Iterative design	• Development & evaluation: qualitative and quantitative testing of usability, principles, and function of the app with voice hearers (details not published).
V-NeST	Software developers; service users; clinicians; technology experts	Not stated	• Development: four development iterations incorporating feedback from clinicians (n = 3), service users (n = 2), and technology experts (n = 2). • Evaluation: pilot study (N = 30), including qualitative interviews on intervention experiences (n = 9).
weCOPE	Service users; practitioners	Not stated	• Development: survey of service users (N = 102) on mobile device use and mHealth interest; focus group with practitioners (N = 6) on attitudes, needs, and potential obstacles. • Prototype testing & evaluation: prototype tested by service users (n = 6) and professionals (n = 6) with think-aloud commentary and usability ratings; pilot study assessing feasibility (n = 9).

This Table includes stakeholder involvement that was explicitly reported as informing intervention design or development. Studies reporting stakeholder involvement without describing impact on design decisions were not included; EIP: early intervention in psychosis; EIS: early intervention in psychosis service; EMA: ecological momentary assessment; FEP: first episode psychosis; LEAP: lived experience advisory panel; MVP: minimum viable product; NHS: national health service; PPI: patient and public involvement; RCT: randomised controlled trial; SAD: social anxiety disorder; SMI: severe mental illness; VA: veterans affairs; VR: virtual reality; YPAG: young person advisory group.

### 3.4 What stakeholders have been involved and how?

Service users were the most frequently involved stakeholder group, participating in the design and development of 27 of the 31 interventions. This was followed by clinicians (n = 20), with comparatively limited involvement of carer and family members (n = 4) and technical and creative contributors (i.e. software developers, engineers, usability experts, graphic designers, and writers) (n = 4). Where sociodemographic data were reported (see Supplementary File 4), the service user samples comprised a majority of men (1586/2767; 57.3%) with gender-diverse identities rarely involved (27/2767; 1%). Reported age data indicated the mean (SD) of stakeholders was 36.01 (8.56). Ethnicity was the least consistently reported demographic characteristic (14/31 interventions). Ethnicity data were available for 1897 service users. Of these, 1237 were white (65.2%), 302 (15.9%) were Black, 48 (2.5%) were Asian, 22 (1.2%) were mixed, and 288 (15.2%) were reported as other ethnic identity. Demographic reporting for clinicians, carers and other stakeholder groups was minimal, with most studies describing professional roles rather than sociodemographic data.

The type of stakeholder involvement and design methods employed varied considerably. Service users contributed at multiple stages of development, from early needs identification to prototype refinement and evaluation. Their involvement included workshops, interviews, surveys, observations, and focus groups shaping intervention content and priorities (n = 23). Many interventions also involved service users in user testing, such as beta testing, think-aloud sessions, and lab-based usability assessments (n = 15). In eight interventions, service users acted as co-designers or content producers, directly contributing avatars, narratives, scenarios, scripts, and language. A minority (n = 7) positioned service users in ongoing advisory roles, such as LEAP or expert reference groups offering feedback and oversight. Fifteen interventions undertook pilot studies to refine and inform further software development.

Other stakeholders contributed complementary perspectives. Clinicians – including psychiatrists, psychologists, nurses, case managers, and mental health staff – were frequently engaged through focus groups, workshops, and advisory teams to inform feasibility, acceptability, and integration with routine care pathways (n = 18). Some clinicians participated directly in prototype testing and co-creation of therapeutic content (n = 7). Carers and family members contributed insights into acceptability and support needs, typically through advisory or focus group formats. Technical and creative contributors often worked iteratively with service users and clinicians to translate lived experience priorities into functional features and therapeutic content (e.g., EMBRACE; Horyzons; STOP; gameChange).

### 3.5 Which DEI-related domains are reported as being addressed in the design of DMHIs for psychosis?

DEI domain coverage reporting was mixed and typically limited across interventions (Table 3, Table 4, Table 5). Forty-three interventions (78.2%) reported addressing at least one DEI-related domain. Thirty interventions (54.5%) reported features spanning at least two of the four overarching domains, including 17 (30.9%) spanning three or more domains and eight (14.5%) spanning all four domains. At the subdomain level, literacy (n = 15), system requirements (n = 16), psychosis-specific adaptations (n = 14), and diverse visual representation (n = 13) were among the most frequently reported considerations. There was limited reporting of DMHI designs addressing cultural adaptation (n = 3), availability in other languages (n = 3), and environmental tailoring to users' physical or community contexts (e.g., safety, walkability, privacy; n = 5). Broader reported coverage across multiple DEI domains was evident for only a small number of interventions, most often those reporting sustained stakeholder engagement, including co-design with people with lived experience and ongoing clinician involvement. Across modalities, explicit framing of intervention design as being driven by equity or inclusion was rarely reported.

**Table 3:** Virtual reality (and related technologies) and DEI domains (n = 19).

	ACCESS				CONTENT					APPEARANCE		STAKEHOLDERS	
Intervention	Internet	Data	Cost	System	Language	Literacy	Env	Disability/ Psychosis symptoms	ID	Diverse rep	Cultural adaptation	EBEs	Others
PhoenixVR													
gameChange													
AVATAR													
Avatar-based therapy													
V-NeST													
CAM+VR													
CBT+AT													
Challenge-VRT													
Modified AVATAR													
VBM													
VR-CBT													
VR-SOAP													
VRETp													
VRT													
Mindfulness-based training in VR													
THRIVE													
VR for PD													
VR CBTp													
VRET + CBT													
**Subdomain totals**	**1**	**0**	**0**	**2**	**0**	**2**	**0**	**2**	**6**	**6**	**0**	**5**	**4**

: DEI-related subdomain reported as addressed. Interventions are ordered by the number of DEI-related subdomains reported, from highest to lowest.

**Table 4:** Blended DMHIs and DEI domains (n = 18).

Intervention	Access				Content					Appearance		Stakeholders	
	Internet	Data	Cost	System	Language	Literacy	Env	Disability and psychosis	ID	Diverse rep	Cultural adaptation	EBEs	Others
Horyzons													
SloMo													
FOCUS													
EMBRACE													
My Journey 3													
Sleep Catcher													
Digital NAVIGATE													
EMPOWER													
HYM													
m-RESIST													
SAVVy													
IMPACHS													
EviBaS													
MUSE-FEP													
CBT2go													
DICE													
SMART													
START													
**Subdomain totals**	**2**	**2**	**4**	**5**	**2**	**7**	**2**	**6**	**2**	**3**	**1**	**11**	**11**

: DEI-related subdomain reported as addressed. Interventions are ordered by the number of DEI-related subdomains reported, from highest to lowest.

**Table 5:** Self-guided digital intervention and DEI framework (n = 18).

Intervention	Access				Content					Appearance		Stakeholders	
	Internet	Data	Cost	System	Language	Literacy	Env	Disability and psychosis	ID	Diverse rep	Cultural adaptation	EBEs	Others
T4RP													
A4i													
Actissist													
ChillTime													
mindLAMP													
NEON													
STOP													
TechCare													
Temstem													
Smartphone app targeting sleep													
weCOPE													
CWV													
HEINS													
MACS													
MCI-S													
HelpID													
Sleepio													
SMARTapp													
**Subdomain totals**	**4**	**3**	**5**	**9**	**1**	**6**	**3**	**6**	**2**	**4**	**2**	**12**	**11**

: DEI-related subdomain reported as addressed. Interventions are ordered by the number of DEI-related subdomains reported, from highest to lowest.

Across the three intervention modalities (Table 3, Table 4, Table 5), VR (and related technologies) demonstrated the lowest overall reported DEI coverage. Within this modality, appearance-related considerations were most frequently reported, with identity-related features and diverse visual representation reported for six of 19 interventions. Involvement of experts by experience was reported for five interventions, whereas access-related considerations were uncommon, including system requirements (n = 2) and internet access (n = 1), with no reported consideration of data or cost. Psychosis-specific tailoring was reported for two interventions, including panic or stop functions (AVATAR), and shortened formats with embedded guidance (VBM). PhoenixVR demonstrated the most extensive reported DEI coverage in this modality, addressing multiple domains including access (offline and home-based use), language accessibility, and identity-neutral design. In contrast, broader equity-related domains (e.g., cultural adaptation, disability access beyond symptoms management, or cost and data considerations) were rarely reported across VR interventions (n = 2).

DEI domains were more frequently reported in blended DMHIs, although coverage remained inconsistent across interventions. Stakeholder involvement was most frequently reported, with both experts by experience and other stakeholders involved in 11 of 18 interventions. Among content-related subdomains, literacy (n = 7) and psychosis- or disability-related tailoring (n = 6) were most frequently reported, while system requirements were reported for five interventions. Cultural adaptation was reported for only one blended intervention. Horyzons had the widest reported coverage across domains, including language, literacy, system access, stakeholder engagement, and cultural adaptation through translation and adaptation across Australia, the U.S.A., and Canada. A broader range of DEI domains was reported for EMPOWER, FOCUS, and SloMo, including lived experience involvement, flexible delivery formats, and attention to accessibility. Within published reports of blended DMHIs, DEI considerations were most often reflected in symptom-relevant content and clinician-mediated support, while explicit design features addressing identity, disability access, or structural barriers such as cost, data burden, or connectivity were less frequently reported.

Stand-alone or minimally supported DMHIs demonstrated the highest overall reported DEI domain coverage across modalities (Table 5), although this coverage was typically selective. Stakeholder involvement was particularly common, with experts by experience reported for 12 of 18 interventions and other stakeholders for 11. System requirements were the most frequently reported access-related subdomain (n = 9), while literacy and psychosis- or disability-related tailoring were each reported for six interventions. Access-related design features (e.g., internet, data, system, or cost considerations) were reported for 13 stand-alone interventions, more frequently than in other modalities, although individual access domains were unevenly represented. Stakeholder involvement, particularly engagement with experts by experience, was commonly reported (n = 13), but was not consistently accompanied by broader reported coverage across access, content, and appearance domains. A small number of interventions demonstrated broader reported coverage across multiple domains; for example, NEON incorporated literacy-aware design, environmental tailoring, psychosis-specific adaptation, diverse visual representation, and lived experience involvement, while T4RP combined system accessibility, cost considerations, psychosis tailoring, and stakeholder engagement. Diverse visual representation was reported in a minority of cases (n = 4), with explicit cultural adaptation or availability in languages beyond English being rarely reported (n = 3).

## 4 Discussion

### 4.1 Main findings

This scoping review examined the design approaches employed in the development of DMHIs for psychosis, the stakeholders involved and design methods used, and the extent to which DEI domains were reported as being addressed during software design. This builds on existing reviews of coproduction in DMHIs (e.g., Brotherdale et al., 2024), by systematically scoping reported tailoring of intervention designs to promote equity and inclusion. Under a quarter of interventions explicitly named a design approach, with user-, person-, or human-centred design most frequently reported. Just over half of the interventions reported some level of stakeholder involvement in their design, most commonly involving people with lived experience of psychosis and clinicians. Most interventions were reported as addressing at least one DEI-related domain, most commonly psychosis-specific tailoring, literacy-aware design, or basic accessibility. However, comprehensive reported coverage across multiple domains of DEI was uncommon. The findings indicate that while design activities are increasingly employed in DMHIs for psychosis, systematic reporting of the application of DEI principles was less evident in published accounts of intervention development.

Reporting of stakeholder sociodemographic characteristics was highly variable, particularly in early-stage design studies. While this may reflect small samples, anonymity concerns, or classification of activities as PPI, limited reporting constrains assessment of whose perspectives inform design. Where reported, samples were predominantly younger and not gender-diverse, while almost two-thirds of stakeholders for whom ethnicity was reported were White, raising concerns about under-representation of populations disproportionately affected by psychosis (Kirkbride et al., 2017; Watson et al., 2022; Barr et al., 2021). This imbalance may narrow the range of cultural and social experiences informing design decisions, potentially limiting the relevance and acceptability of resulting interventions across diverse populations (Brotherdale et al., 2024). Moving beyond convenience sampling toward purposive recruitment of individuals with intersecting marginalised identities, especially at early design stages, can support equity, as well as more effective use of limited design resources (Palinkas et al., 2015).

The uneven coverage of DEI considerations observed in this review is consistent with wider digital mental health research highlighting gaps in cultural adaptation, demographic representation, and attention to equity-related characteristics (Spanhel et al., 2021; Ellis et al., 2022; Hall et al., 2026). In the present review, accessibility-related considerations, including literacy, system requirements, and adaptations addressing cognitive and psychosis-related needs, were relatively prominent, whereas cultural adaptation and wider sociocultural dimensions of inclusion were less frequently reported. This is notable given emerging evidence supporting the acceptability and potential effectiveness of culturally adapted DMHIs (Ellis et al., 2022). Cultural adaptation may also be constrained by the additional time and resources required for community engagement, translation, and iterative development across different populations and contexts (Sangraula et al., 2021; Alnaghaimshi et al., 2026). Structural and practical barriers to participation among minoritised populations in mental health research, including language, recruitment processes, and resource constraints, may further contribute to these gaps by limiting whose perspectives inform intervention development (Brown et al., 2014). These findings underscore the importance of considering multiple and intersecting dimensions of inclusion in DMHI design, as addressing particular accessibility needs or involving people with lived experience is not sufficient to ensure inclusion.

A consistent pattern in the published literature was that interventions reporting more sustained stakeholder engagement were more likely to address a wider range of DEI domains. Interventions reporting lived experience input across multiple stages – progressing beyond consultation towards early and sustained stakeholder involvement – were more likely to address a wider range of DEI domains. However, addressing DEI domains was rarely articulated as an explicit design aim in published reports, with DEI-related features often described within broader participatory design activities. These findings suggest that whilst participatory design practices may support aspects of inclusive design, limited explicit use of DEI-focused frameworks is accompanied by uneven reported coverage across relevant domains (Ramos et al., 2021; Harrington et al., 2019a). Future DMHI development would benefit from drawing on structured approaches, such as the adapted Culturally Informed Design Framework that guided this review (Valdez et al., 2012; Ramos et al., 2021; Figueroa et al., 2024).

Our findings suggest that many DEI-relevant considerations are either reported only at later stages of development or described implicitly, despite their clear relevance to implementation success (Aref-Adib et al., 2019). This is notable in the context of the persistent evidence-practice gap in digital health (Theobald et al., 2018). Implementation requirements should therefore be considered during development rather than treated as downstream concerns. Embedding DEI considerations early in design may help anticipate barriers related to usability, accessibility, and equity, thereby strengthening readiness for scale-up. The DEI domains considered in this review align closely to criteria within implementation frameworks such as the UK's NICE Evidence Standards Framework for Digital Health Technologies (National Institute for Health and Care Excellence, 2022a, National Institute for Health and Care Excellence, 2022b), which requires attention to user involvement, usability, and health inequalities. Design processes and outputs that make these considerations explicit may therefore function not only as inclusive practices but as facilitators of adoption.

Although this review was not designed to assess whether DEI-informed design is associated with improved clinical or engagement outcomes, the absence of causal evidence should not be interpreted as grounds for deprioritising inclusion. Nonetheless, inclusive design alone is unlikely to be sufficient to ensure sustained engagement. While DEI-informed design supports equitable access and participation, continued use also depends on broader user experience factors, including usability, perceived usefulness, and appeal (Norman, 2024; Eisner et al., 2025). These factors are integral to robust design processes; however explicit attention to DEI domains does not necessarily guarantee an intervention will be experienced as relevant or valuable in practice (Smith et al., 2024). This distinction is particularly important in psychosis, where problems with motivation, information processing, and trust in digital technology are common.

Interpretation of DEI coverage also requires consideration of intervention modality and delivery context. For clinician-supported interventions, particularly VR-based therapies, domains such as internet connectivity or system requirements may appear less salient during early development because access is mediated through a core team. However, as interventions move toward scale-up and routine delivery, these factors can become critical determinants of equitable implementation and sustainability (Greenhalgh et al., 2017). Explicitly addressing such assumptions during design is therefore essential to avoid introducing barriers for individuals with limited digital access or those in under-resourced settings (Torous et al., 2025a, b).

The available evidence was heavily concentrated in high-income countries, with minimal representation from low- and middle-income countries (LMICs). This geographical skew has important equity implications given global disparities in mental health care and digital infrastructure (Carter et al., 2021). Without culturally informed adaptation, deploying interventions developed in high-income contexts elsewhere risks poor contextual fit and reduced effectiveness (Hall et al., 2016; Chau et al., 2025). The implementation context for DMHIs may also differ substantially between HICs and LMICs, resulting in different DEI priorities. For example, system requirements, device availability, connectivity, and data costs may assume greater importance in settings where digital infrastructure and access are constrained. At the same time, the widespread adoption of mobile technologies presents opportunities to extend mental health support in settings where access to specialist care is limited (Merchant et al., 2020; Fu et al., 2020). Future research should therefore prioritise co-designed adaptation and development within diverse global contexts, responsive to locally relevant cultural, technological, and health-system priorities.

### 4.2 Limitations

DEI-related design features were often poorly reported, requiring reliance on narrative descriptions. As only explicitly stated features were coded, DEI coverage may have been underestimated where relevant considerations were implicit or undocumented (e.g., cost-free access). While direct examination of interventions may have revealed additional features, transparent reporting remains essential for appraisal and replication. Accordingly, where DEI-related features were not reported, this should be interpreted as absence of evidence rather than evidence that these considerations were absent from intervention development. Data extraction and coding were also conducted by a single reviewer, which may have increased the risk of extraction errors or relevant design features being overlooked. Although the extraction framework was iteratively refined in consultation with the wider research team, independent duplicate extraction would have strengthened the reliability of the review.

The review was restricted to peer-reviewed, English-language publications, which may have excluded relevant interventions developed or evaluated in low- and middle-income countries or reported in grey literature. As early-stage design work is increasingly disseminated through non-traditional, open science outputs, our approach, while ensuring feasibility and consistency, may have reinforced existing publication and geographical biases.

Finally, although design features were coded across multiple DEI domains where relevant, the use of a structured framework necessarily involved abstracting complex and interrelated design practices into analytic categories. For instance, design features such as simplified language combined with culturally relevant content may address literacy, cognitive accessibility, and cultural relevance simultaneously, yet must be analytically separated when mapped to DEI domains. Although predefined subdomains supported consistency in coding, distinctions between categories were not always clear-cut and some classifications therefore required interpretative judgement. While this enabled systematic comparison across interventions, it cannot fully capture the relational, contextual, and intersectional nature of inclusion as enacted within real-world design processes.

### 4.3 Conclusions

Stakeholder engagement was commonly reported in DMHI development, particularly involvement of people with lived experience and clinicians. However, published accounts demonstrated limited comprehensive coverage across DEI domains, including among interventions reporting sustained and in-depth stakeholder involvement. Inclusive design is recommended as a core ethical and methodological requirement for developing equitable digital mental health care for psychosis (WHO, 2019, WHO, 2022). DMHI development studies would benefit from greater transparency in reporting design decisions, purposive stakeholder recruitment to ensure inclusive representation, stakeholder involvement throughout all stages of design and development, and routine use of structured frameworks to guide DEI-informed design across the intervention lifecycle.

## Ethical approval

N/A.

## Funding

The first author (TG) is in receipt of a PhD studentship funded by the National Institute for Health and Care Research (NIHR) Maudsley Biomedical Research Centre (BRC). AH and TW acknowledge funding from the Maudsley Biomedical Research Centre at South London and Maudsley NHS Foundation Trust and 10.13039/501100000764King's College London.

## Declaration of competing interest

The authors declare the following financial interests/personal relationships which may be considered as potential competing interests: Thomas Gant reports financial support and article publishing charges were provided by National Institute for Health Research Maudsley Biomedical Research Centre. Amy Hardy reports financial support was provided by National Institute for Health Research Maudsley Biomedical Research Centre. Thomas Ward reports financial support was provided by National Institute for Health Research Maudsley Biomedical Research Centre. AH, PG, and TW are co-founders of SloMo therapy. TW and AH are co-founders of AVATAR therapy. If there are other authors, they declare that they have no known competing financial interests or personal relationships that could have appeared to influence the work reported in this paper. AH, PG, and TW are co-founders of SloMo therapy. TW and AH are co-founders of AVATAR therapy.
